# Modulation of the Host Interferon Response and ISGylation Pathway by *B. pertussis* Filamentous Hemagglutinin

**DOI:** 10.1371/journal.pone.0027535

**Published:** 2011-11-30

**Authors:** Christine Dieterich, David A. Relman

**Affiliations:** 1 Department of Microbiology and Immunology, Stanford University School of Medicine, Stanford, California, United States of America; 2 Department of Medicine, Stanford University School of Medicine, Stanford, California, United States of America; 3 Veterans Affairs Palo Alto Health Care System, Palo Alto, California, United States of America; Indian Institute of Science, India

## Abstract

*Bordetella pertussis* filamentous hemagglutinin (FHA) is a surface-associated and secreted protein that serves as a crucial adherence factor, and displays immunomodulatory activity in human peripheral blood mononuclear cells (PBMCs). In order to appreciate more fully the role of secreted FHA in pathogenesis, we analyzed FHA-induced changes in genome-wide transcript abundance in human PBMCs. Among the 683 known unique genes with greater than 3-fold change in transcript abundance following FHA treatment, 125 (18.3%) were identified as interferon (IFN)-regulated. Among the latter group were genes encoding several members of the IFN type I response, as well as 3 key components of the ISGylation pathway. Using real-time RT-PCR, we confirmed FHA-associated increases in transcript abundance for the genes encoding ubiquitin-like protein, ISG15, and its specific protease USP18. Western-blot analysis demonstrated the presence of both, free ISG15 and several ISGylated conjugates in FHA-stimulated PBMC lysates, but not in unstimulated cells. Intracellular FACS analysis provided evidence that monocytes and a natural killer-enriched cell population were the primary producers of ISG15 in PBMCs after FHA stimulation. Our data reveal previously-unrecognized effects of *B. pertussis* FHA on host IFN and ISGylation responses, and suggest previously-unsuspected mechanisms by which FHA may alter the outcome of the host-pathogen interaction.

## Introduction

*Bordetella pertussis* is a human restricted pathogen and the causative agent of the acute respiratory disease, pertussis or whooping cough. Despite the use of an effective vaccine since the 1940s, pertussis remains a major cause of childhood mortality worldwide and has re-emerged in some highly vaccinated populations [1], [2]. *B. pertussis* colonizes the upper respiratory tract; after attachment to the cilia of epithelial cells, it proliferates on the ciliated mucosal surface, resulting in damage to the mucosa, influx of inflammatory cells, and the shedding of cells into the lumen of the respiratory tract. *B. pertussis* is primarily an extracellular organism, but it may persist within leukocytes and epithelial cells [3], [4]. The infection process is mediated by several virulence factors [5], most of which are tightly regulated by a two-component signal transduction system, BvgAS [6].

A key BvgAS-regulated virulence factor is filamentous hemagglutinin (FHA), which plays a crucial role in mediating adherence to eukaryotic cells [7]. Because of its immunogenicity and immunoprotective activity, FHA is a component of most acellular pertussis vaccines. FHA, encoded by *fhaB*, is synthesized as a 367 kDa precursor, FhaB, which undergoes extensive N- and C-terminal modifications to form the mature 220 kDa FHA protein. After transport across the cytoplasmic membrane by a Sec signal peptide-dependent pathway, FhaB is secreted across the outer membrane by the two-partner secretion system. During the translocation process, approximately 130 kDa of the C terminus is proteolytically cleaved by SphB1. While SphB1 contributes to FHA release into the extracellular milieu, it is not required, and the mechanism by which FHA is released from the cell surface remains unclear [8]. Another important unanswered question addressed by this work in particular, concerns the role of the released form of FHA in pathogenesis [8].

*In vitro* studies have suggested that FHA functions as an adhesin and several binding domains have been identified: an Arg-Gly-Asp (RGD) triplet [9], a carbohydrate recognition domain (CRD) for binding to ciliated respiratory epithelial cells and macrophages [10], and a lectin-like domain for binding to heparin and other sulphated carbohydrates on non-epithelial cells [11]. FHA also exhibits several immunomodulatory properties, including its ability to interfere with NF-κB activation [12] and to induce the secretion of both pro- and anti-inflammatory cytokines by macrophages [13], [14].

The role of FHA *in vivo* has not been entirely elucidated, primarily because a natural nonhuman host for *B. pertussis* does not exist, but also because of the complexity of this molecule and its associated biological activities. In rabbit and mouse models of *B. pertussis* infection, there are conflicting results regarding the role of FHA in the persistence of bacteria in the lung. However, in a rat model of natural *B. bronchiseptica* respiratory infection, FHA was absolutely necessary, although not sufficient, for tracheal colonization [15]. The secreted form of FHA might facilitate dispersal of bacteria from microcolonies and detachment from epithelial surfaces, and thereby promote bacterial spread [16]. In addition, *B. bronchiseptica* FHA may control the repertoire or abundance of cytokines induced in the lungs of infected mice, as suggested by the substantial inflammation induced by an *fhaB* null mutant strain, in contrast to the mild inflammation induced by wild-type *B. bronchiseptica* [17]. Because of the recently demonstrated functional interchangeability of *B. pertussis* and *B. bronchiseptica* FHA [18], the above findings are likely to apply to *B. pertussis* FHA as well.

The analysis of genome-wide host transcriptional responses is one approach for exploring and further characterizing complex host-pathogen interactions. During the past decade, this approach has been successfully employed to study the host response to a broad range of pathogens and virulence factors [19]. Although exposure to pathogens induces a broadly conserved host cell transcriptional program [20], [21], specific profiles have been observed for individual virulence factors, perhaps because different microbial products are detected by different combinations of receptors, such as Toll-like receptors (TLRs) [22]. Activation of TLRs leads to the production of various cytokines and chemokines, including type I interferons (IFNs). Type I IFNs (IFN-α, IFN-β, IFN-ε, IFN-κ, and IFN-ω) are potent immunoregulators, responsible for activating key components of the innate and adaptive immune system. IFN-induced activation of the JAK/STAT pathway triggers the transcription of hundreds of interferon stimulated genes (ISGs). ISG15, one of the earliest and most strongly induced ISGs, is the oldest known member of the ubiquitin-like (UbL) modifier polypeptides and was described more than 30 years ago [23]. However, it is only recently that interest has focused on understanding this UbL polypeptide with the unique properties of acting both as a modifier of protein function and as a cytokine that modulates immune responses [24]. ISGylation, the process by which ISG15 is conjugated to a target protein, involves a set of enzymes analogous to the ubiquitin modification system: the ubiquitin activating enzyme E1-like (UBE1L), the E2 conjugating enzyme UBC8, the putative ISG15 E3 ligase, and the ISG15 deconjugating enzyme UBP43 (USP18) [25]. Several hundreds of target proteins have been identified [26]–[28]. Recently, a novel role of ISG15 in protecting cells from infection by several viruses has become evident [29]–[33]. The role of ISG15 and the ISGylation pathway in defence against bacterial infection has not been described in detail but it is of great interest due to the fact that 1) ISG15 is induced upon contact with bacteria or bacterial products [20], [34], [35] and 2) type I IFNs play an important role in the host response to bacterial infections [36].

The goal of this study was to characterize the effects of purified *B. pertussis* FHA on human cells by examining the genome-wide transcriptional response of human peripheral blood mononuclear cells (PBMCs). Such an investigation might enhance our understanding of the contributions of free, soluble FHA in the pathogenesis of Bordetella infection. One would expect resident and recruited mucosal mononuclear leukocytes to encounter secreted forms of FHA during the course of disease. We demonstrate that FHA is a strong inducer of the IFN response, including IFN type I. In addition, purified FHA enhances expression of at least three members of the ISGylation pathway: ISG15, USP18, and UBE1L. These results suggest new putative mechanisms by which this key bacterial adhesin modulates the host immune response, mechanisms that may be relevant both in the context of natural infection and following administration of FHA-containing *Bordetella* vaccines.

## Results

### Genome-wide responses of human PBMCs to purified *B. pertussis* FHA

To gain insight into the effect of cell-free FHA on host responses, we characterized the genome-wide gene expression program in human PBMCs stimulated with purified FHA. This analysis was undertaken with four different FHA preparations, purified from four different *B. pertussis* strains (two clinical isolates and two lab strains), in order to recognize conserved activities associated with this protein and avoid strain-specific biases. Prior to FHA preparation, all genes associated with pertussis toxin (PT) expression were deleted from the strains, so that PT co-purification would be eliminated. One of the preparations, FHA-1, had been used previously to study the immunological properties of FHA [12], [13]. While the presence of trace amounts of non-FHA contaminants in the preparations could not be ruled out, even after verification of the purity by Coomassie blue staining of protein-loaded acrylamide gels and Western immunoblot analysis (Fig. S1), the use of several independent preparations with presumably different contaminants in different, albeit low amounts, greatly strengthened the likelihood that the dominant, conserved activities and responses elicited by these preparations are attributable to FHA.

**Figure 1:**
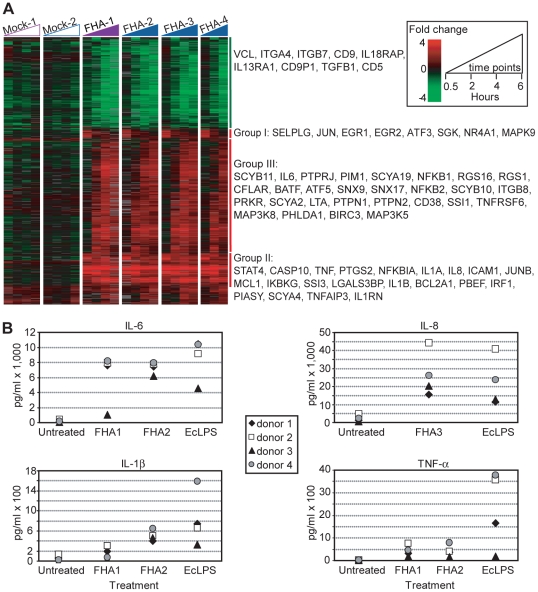
Overview of gene expression and pro-inflammatory cytokine response in FHA-treated PBMCs. (A) PBMCs were stimulated for 0.5, 2, 4, and 6 hrs (time course represented by the triangles) with FHA-1, FHA-2, and FHA-3 at 5 µg/ml, and for 0.5, 2, and 6 hrs with FHA-4 at the same concentration. As a control, cells were treated with similar volumes of the corresponding elution buffers (“Mock-1” and “Mock-2”). A total of 1,235 elements displayed a ≥3-fold change in transcript abundance in at least 3 arrays; they are represented in a hierarchical cluster format. Data from individual elements or genes are represented as a single row, and different time points in the time courses are shown as columns. Red and green denote expression levels greater than or less than, respectively, baseline values (average of four untreated samples taken at time zero). The intensity of the color reflects the magnitude of the change from baseline. Gray color represents missing or excluded data. (B) PBMCs, isolated from four healthy individuals (donors 1–4), were stimulated with FHA (FHA-1, -2, or -3) at 5 µg/ml or EcLPS at 1 µg/ml, or were untreated. After 8 hrs (IL-8) and 24 hrs (TNF-α, IL-1β, and IL-6) of exposure, the supernatants were collected and analyzed. Data were obtained from two independent experiments, one experiment in which cells were treated for 8 hrs and one experiment in which cells were treated for 24 hrs.

Cultures of 3×10^6^ PBMCs were stimulated for 0.5, 2, 4, and 6 hours with 5 µg/ml of each purified *B. pertussis* FHA preparation. At that concentration, FHA-1 is known to induce both pro-inflammatory and pro-apoptotic responses in human cells [13] and to modulate the NF-κB pathway [12]. Control PBMC cultures were simultaneously treated with similar volumes of elution buffers (“Mock-1” and “Mock-2”), and sampled at the same time points. A total of 23 samples were analysed, representing a total of 23 arrays. Among the 25,867 microarray elements whose hybridization signals were measured with confidence (Materials & Methods), 1,235 elements exhibited at least a 3-fold change in transcript abundance relative to the untreated samples in at least 3 out of 23 arrays. (The microarray data from the FHA-4 stimulated cells at the 4 h time point were not included in the analysis because of poor microarray signal quality.) The 683 known unique genes represented by these 1,235 elements were considered to be FHA-responsive and were organized by hierarchical clustering (Fig. 1A). All four FHA preparations induced very similar gene expression profiles, suggesting minimal functional differences between FHA protein from different *B. pertussis* strain backgrounds.

FHA-responsive genes could be assigned to two major classes: an FHA-activated cluster comprising 817 elements (419 known unique genes; Table S1) and a FHA-repressed cluster comprising 418 elements (254 known unique genes; Table S2). The genes in the activation cluster could be further subdivided into three groups, according to their temporal expression profile (Fig. 1A). Group I contained genes whose expression was strongly up-regulated at 0.5 hour, but was lower at subsequent time points. Group II included genes whose expression was induced at 0.5 hour and remained elevated throughout the experiment. In Group III, the largest group, up-regulation occurred later, starting at 2 or 4 hours after FHA treatment.

Several transcription factors and regulators of transcription, including JUN, JUNB, EGR, MAPK9, IRF1, STAT4, and ATF3, were induced within 30 minutes of FHA exposure. Members of the NF-κB pathway, including the transcriptional regulators NFKB1 and NFKB2 and the inhibitor NFKBIA, were also up-regulated by FHA treatment. As expected, the expression of several targets of this pathway was also increased, including genes encoding cytokines (TNF, IL1A, IL1B, IL6, and LTA), chemokines (IL8, SCYA2/CCL2, and SCYB11/CCL11), adhesion molecules (ICAM1), pro-inflammatory enzymes (PTGS2/COX2), and kinases (PRKR/EIF2AK2, IKBKG, MAP3K8).

Genes encoding cytokines and chemokines were some of the most strongly FHA-induced loci: among the 20 unique genes up-regulated at least 16-fold compared to unstimulated cells, 8 encoded proteins that belonged to this category (data not shown). This result suggested that FHA is a strong pro-inflammatory stimulus, and was confirmed by measuring the levels of TNF-α, IL-1β, IL-6, and IL-8 protein in the supernatant of FHA-exposed PBMCs (Fig. 1B). We stimulated 2×10^6^ cells from each of 4 different healthy donors, with 5 µg/ml FHA (FHA-1, FHA-2, or FHA-3) or 1 µg/ml *E. coli* lipopolysaccharide (EcLPS) for 8 or 24 hours. We confirmed increased levels of TNF-α (401±82 and 2±0 pg/ml for FHA-treated [n = 8] and mock-treated [n = 4] samples, respectively), IL-1β (325±74 and 49±28 pg/ml for FHA-treated [n = 8] and mock-treated [n = 4] samples, respectively), IL-8 (26,292±6,243 and 1,948±898 pg/ml for FHA-treated [n = 4] and mock-treated [n = 4] samples, respectively), and IL-6 (6,678±844 and 119±78 pg/ml for FHA-treated [n = 8] and mock-treated [n = 4] samples, respectively), but noted some inter-donor variation (results are given as average ± SEM).

Following FHA exposure, genes involved in the negative regulation of the inflammatory response through the JAK/STAT pathway, such as SSI/SOCS, PIASY/PIAS4, and PTPs, were induced. Regulation of the inflammatory response by FHA may also occur through changes in expression of cytokine receptors, as revealed by down-regulation of genes encoding cytokine receptors IL13RA1 and IL18RAP and up-regulation of IL1 receptor antagonist IL1RN.

### FHA induces a host response distinct from that induced by *B. pertussis* or its LPS

**Figure 2:**
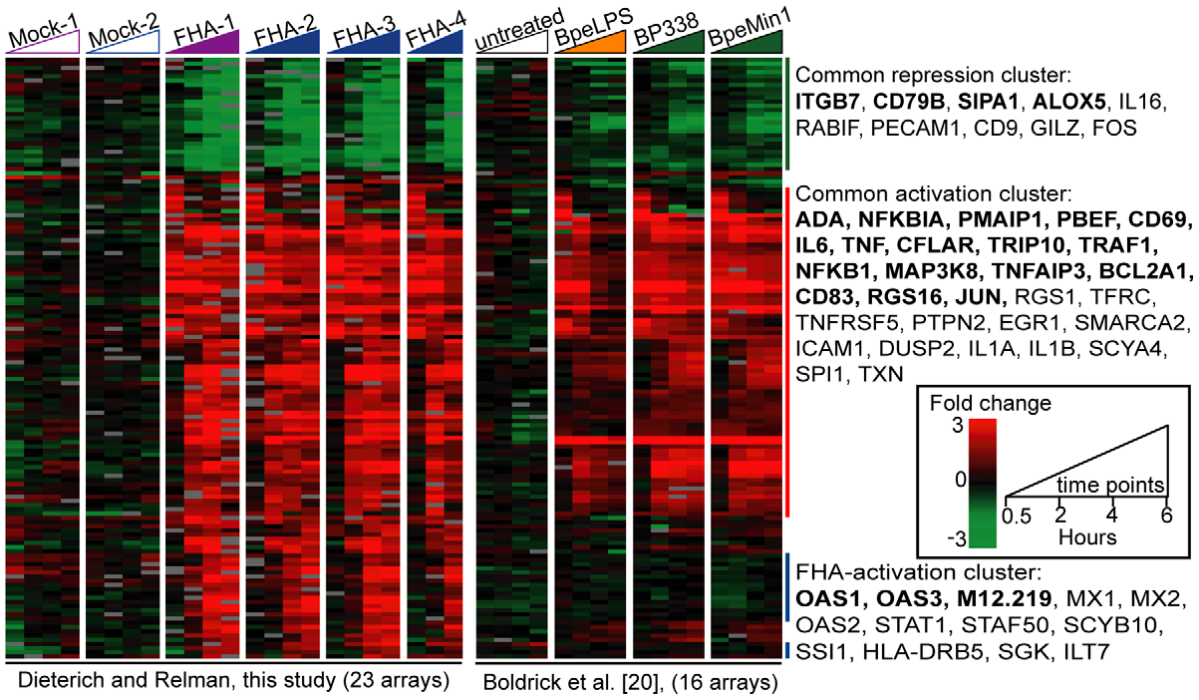
FHA induces a distinct response as compared to heat-killed *B. pertussis* and its purified LPS. Data for the 1,235 FHA-responsive elements from our 23 arrays were compared to data for the 920 elements previously described by Boldrick *et al.* from 16 arrays [20], whose expression in PBMCs displayed a ≥2.5-fold change from baseline (*t* = 0) in response to BpLPS (1 µg/ml) or to two heat-killed *B. pertussis* strains (BP338 and Minnesota 1 [BpeMin1]). The 143 elements common to both data sets are represented in a hierarchical cluster format. Each time course is represented by a triangle at the top of the Figure; the times of exposure are 0.5, 2, 4, and 6 hrs for all conditions except for FHA-4, in which the 4 hour time point is missing. The genes represented by at least two non-identical elements on the arrays are indicated in bold.

We evaluated the specificity of these gene expression responses to FHA, and whether some responses might be attributable to LPS. We compared our data (23 arrays, 1,235 elements) to previously-published gene expression profiles from human PBMCs exposed to heat-inactivated *B. pertussis* (strains BP338 and Minnesota 1 [BpeMin1], ∼1 bacterium per cell) or 1 µg/ml *B. pertussis* LPS (BpeLPS) (16 arrays, 920 elements) [20]. The use of identical strains and microarray platforms facilitated this comparative analysis. A total of 143 elements (64 known unique genes) with varying transcript abundance were identified in both our 1,235 and Boldrick's 920 elements; they are represented in Fig. 2, and organized by hierarchical clustering. Most of these genes were similarly regulated (both up and down) in all treated samples. The 30 genes in the common activation cluster encode cytokines and chemokines, adhesion molecules, transcription factors/regulators, metabolism and signalling proteins, regulators of apoptosis, and lymphocyte activation proteins. A total of 13 genes were activated by FHA (“FHA activation cluster”) and not by heat-killed *B. pertussis* and LPS. This cluster was composed primarily (9/13, 69%) of interferon (IFN)-stimulated genes: MX1, MX2, OAS1, OAS2, OAS3, STAT1, STAF50/TRIM22, SCYB10/CXCL10, and SSI1/SOCS1.

### IFN responses dominate FHA-associated expression profiles

We compared the 1,235 FHA-responsive elements presenting ≥3-fold change in transcript abundance relative to the untreated samples with lists of known IFN-α-, -β-, and -γ-regulated genes [36]–[40], in order to obtain a more complete transcript-associated profile of the FHA-induced IFN response. This approach identified 282 IFN-regulated elements (Table S3), representing a total of 125 known unique genes, and suggested that FHA is a strong inducer of the IFN type I response. FHA stimulated increased levels of transcripts for genes associated with three well-characterized IFN-induced antiviral mechanisms: the dsRNA-activated protein kinase R (PRKR/EIF2AK2), the 2′-5′-oligoadenylate synthases (OAS), and the myxovirus resistance (MX) proteins [40]. FHA also increased transcript levels for other genes that encode proteins with potentially important antiviral activities such as ISG20, guanylate-binding protein 1 (GBP1), promyelocytic leukemia protein (PML), RNA-specific adenosine deaminase 1 (ADAR), and the adenosine deaminases (ADA).

### FHA activates the host ISGylation pathway

The FHA-induced IFN type I response featured increased transcript levels for genes encoding three key members of the ISGylation pathway: ISG15, USP18, and UBE1L. ISG15, a ubiquitin-like protein, is one of the IFN-stimulated genes (ISGs) induced most rapidly and strongly by type I IFNs, as well as by viral infection and LPS [24]. USP18, also known as UBP43, is an ISG15-specific protease, removing ISG15 from target proteins [41] and is a newly identified regulator of the IFN response [42]. Transcript levels for UBE1L, a gene that encodes the ISG15-activating enzyme [43], increased ≥2.5-fold over levels in untreated cells (data not shown).

**Figure 3:**
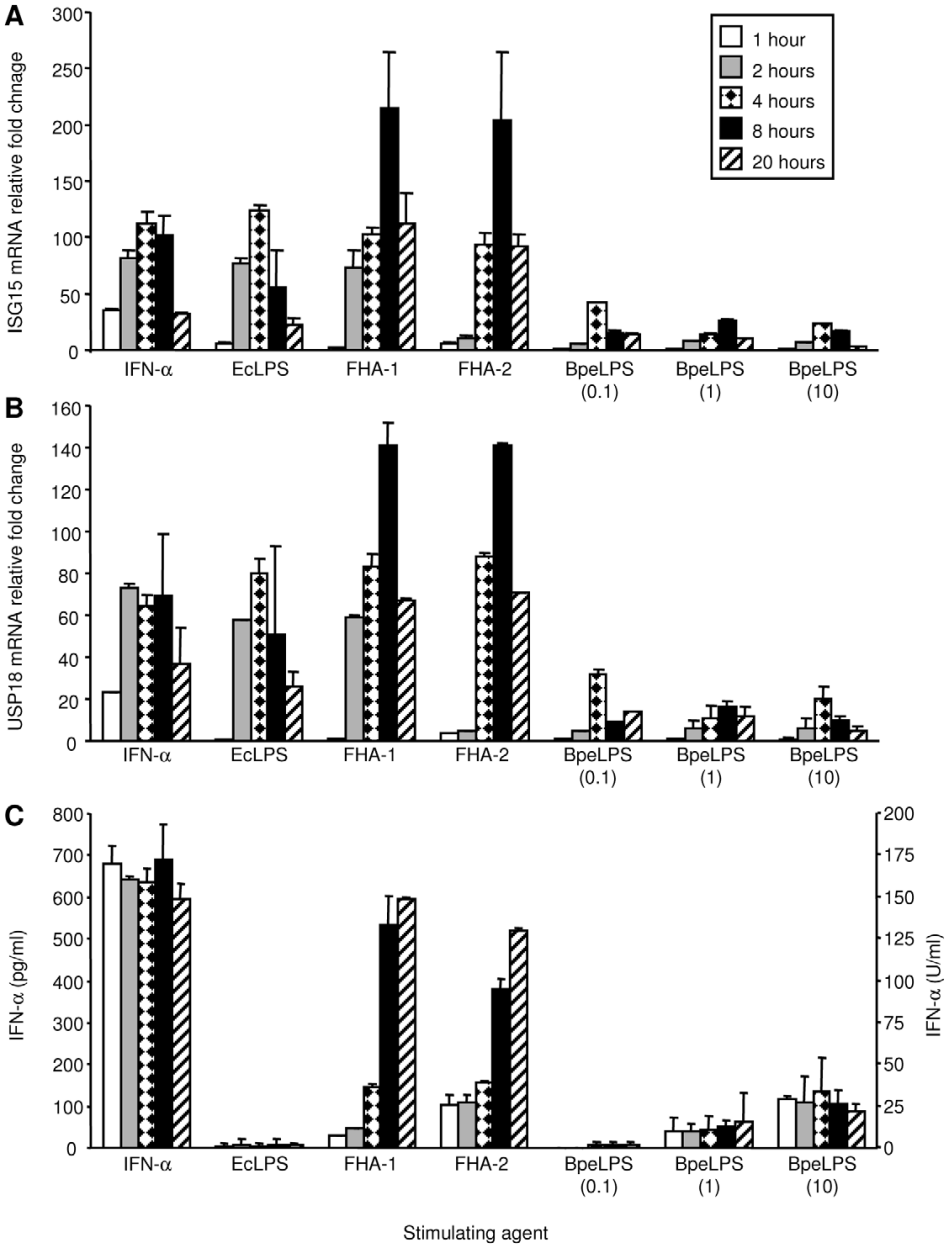
ISG15 and USP18 transcript abundance and levels of secreted IFN-α protein in PBMCs. PBMCs were stimulated with IFN-α at 1,000 U/ml, EcLPS at 1 µg/ml, FHA-1 and FHA-2 at 5 µg/ml, or BpeLPS at 0.1, 1, or 10 µg/ml for 1, 2, 4, 8, and 20 hrs (1 well/condition except for untreated cells at time 0 for which 3 wells were used). After each time point, total RNA was collected and analyzed with RT-PCR using primers specific for ISG15 (A) or USP18 (B). The fold transcript abundance change is calculated relative to levels occurring in untreated cells at the beginning of the time course, and the average and range of fold change are displayed. The cell supernatants were collected and evaluated for the presence of IFN-α protein using ELISA (C). IFN-α concentration was measured in pg/ml (left scale bar) and converted to U/ml (right scale bar) using the conversion factor of ∼4 pg/unit, according to the manufacturer's instructions. Each sample was assayed twice, and the standard deviation was calculated based on the average value of the two independent measurements.

The increased abundance of ISG15 and USP18 transcripts was validated by RT-PCR with the same bulk RNA used for the microarray analysis (data not shown). ISG15 and USP18 expression levels were further validated in an independent experiment: PBMCs were stimulated for 1, 2, 4, 8, and 20 hrs with 1,000 units (U)/ml IFN-α, 1 µg/ml EcLPS, 5 µg/ml FHA (FHA-1 and FHA-2), or 0.1, 1, and 10 µg/ml BpeLPS. At each time point, the culture supernatant was harvested to assess the presence of IFN-α by ELISA, and the cells were collected to perform RT-PCR with primers specific for ISG15 and USP18 (Fig. 3). FHA treatment induced the production of IFN-α as early as 1 hr after exposure. Peak concentrations of IFN-α after FHA stimulation were higher than after treatment with 10 µg/ml BpeLPS (595±3 vs. 135±82 pg/ml after stimulation with FHA and BpeLPS respectively) (Fig. 3C). Increased ISG15 transcript abundance was detected 2 to 4 hrs after FHA treatment (depending on the FHA preparation), with peak expression at 8 hours post-treatment (Fig. 3A). In cells stimulated with 1,000 U/ml IFN-α, the increase in ISG15 transcript abundance began earlier and was measurable (36±1 –fold change relative to untreated cells at time zero) after 1 hr of treatment. Similar patterns were found for expression of USP18 (Fig. 3B). Comparable results and similar kinetics were reproduced in an independent experiment using differing concentrations (1, 5, and 10 µg/ml) of FHA (data not shown).

Enhanced ISG15 and USP18 expression might be solely attributable to FHA-induced IFN-α release. However, our data suggest that FHA might also stimulate expression of these two genes through a mechanism independent of IFN-α, similar to the genotoxic agent, camptothecin which activates ISG15 expression in a p53-dependent, and IFN- and Jak-Stat-independent manner [44]. Two arguments support this hypothesis. First, at 8 and 20 hrs post-treatment, IFN-α concentrations were similar in the supernatants of FHA- and IFN-α-treated cells (Fig. 3C). However, at the same time points, relative levels of ISG15 and USP18 transcripts were ≥1.8-fold higher in FHA-treated cells than in IFN-α-treated cells. Similarly, at 4 hrs post-treatment, relative levels of ISG15 and USP18 transcripts were similar in FHA- and IFN-α-treated cells, even though secreted IFN-α levels were four times lower in FHA-treated cells. Second, although 5 µg/ml FHA and 10 µg/ml BpeLPS induced similar levels of IFN-α between 1 and 4 hrs post-treatment (Fig. 3C), FHA was associated with 4-fold higher levels of ISG15 and USP18 transcripts (Figs. 3A, 3B). This observation was confirmed in two other independent experiments (data not shown). To rule out the possibility that BpeLPS had an inhibitory effect on ISG15 and USP18 expression, BpeLPS (1 and 10 µg/ml) was added to 5 µg/ml FHA, with no effect on ISG15 or USP18 expression levels (data not shown).

### FHA induces the production of free ISG15 and several ISGylated conjugates

Up-regulation of the ISGylation pathway, especially in the context of a bacterial infection, is a relatively recent finding that has not been well-characterized. We focused on our observation that *B. pertussis* FHA affects the transcription of several members of the ISGylation pathway, and in particular on ISG15, the component of this pathway that becomes conjugated to cellular target proteins [26] and that is associated with cytokine-like immunomodulatory activities [45], [46].

**Figure 4:**
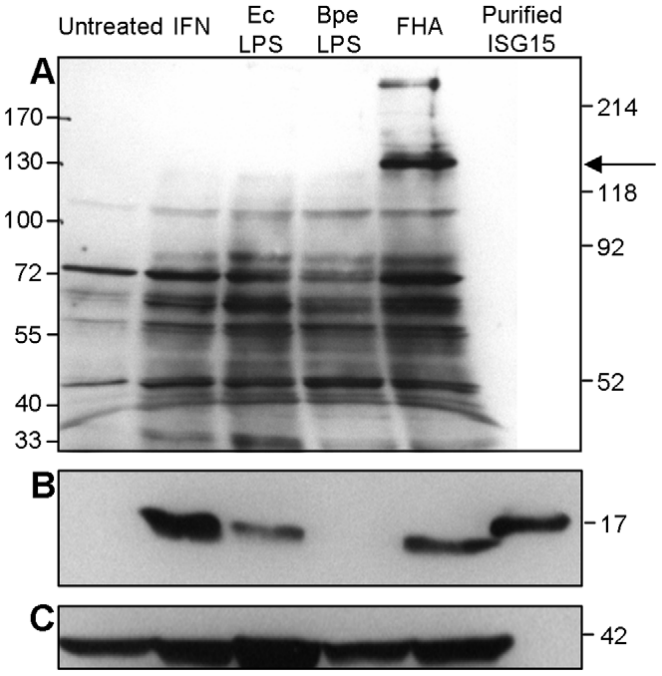
Expression of ISG15 and ISGylated proteins in PBMCs. PBMCs were stimulated with IFN-α (IFN) at 1,000 U/ml, EcLPS at 1 µg/ml, BpeLPS at 10 µg/ml, or FHA-2 (FHA) at 5 µg/ml or remained untreated for 20 hrs. Cell lysates were prepared as described in Materials & Methods, and 90 µg of total protein was resolved with 10% or 15% SDS-PAGE under reducing conditions. Immunoblotting was performed with rabbit polyclonal anti-ISG15 to reveal ISG15 conjugates (A, 10% SDS-PAGE gel) and mouse monoclonal anti-ISG15 clone 2.1 to reveal free ISG15 (B, 15% SDS-PAGE gel). The latter membrane was re-probed with anti-actin antibody to assess protein loading (C). Where indicated, 20 ng purified ISG15 was resolved. Relative mobility of molecular weight markers is indicated to the left of the figure. The arrow points to an ISGylated protein of ∼130 kDa in FHA-treated cells.

We first examined whether the increase in ISG15 transcript abundance correlated with an increase in ISG15 and ISGylated protein levels. PBMCs (5×10^7^) were stimulated with 1,000 U/ml IFN-α, 1 µg/ml EcLPS, 10 µg/ml BpeLPS, or 5 µg/ml FHA-2. After 20 hrs incubation, intracellular ISGylated proteins (Fig. 4A) and free ISG15 (Fig. 4B) were detected by Western immunoblot analysis using rabbit polyclonal and mouse monoclonal antibodies, respectively. Compared to untreated cells, FHA-treated cells produced larger quantities of ISGylated protein and free ISG15, and similar quantities to those found in IFN- and EcLPS-treated cells. No free ISG15 was detected in cells stimulated with BpeLPS, although we cannot rule out the possibility that some ISG15 protein might be expressed in both unstimulated and BpeLPS-stimulated cells and would have been detected with additional amounts of loaded protein. FHA-treated cells contained an additional ISGylated protein, of ∼130 kDa, not detected in cells treated with LPS or IFN-α (Fig. 4A, arrow). This result was reproduced in PBMCs isolated from two different healthy donors in two independent experiments (data not shown). Efforts to identify this ∼130 kDa ISGylated protein(s) using mass spectrometry failed to provide a specific result (data not shown).

### Monocytes and NK-enriched cell populations are the major producers of ISG15 after FHA stimulation of PBMCs

**Figure 5:**
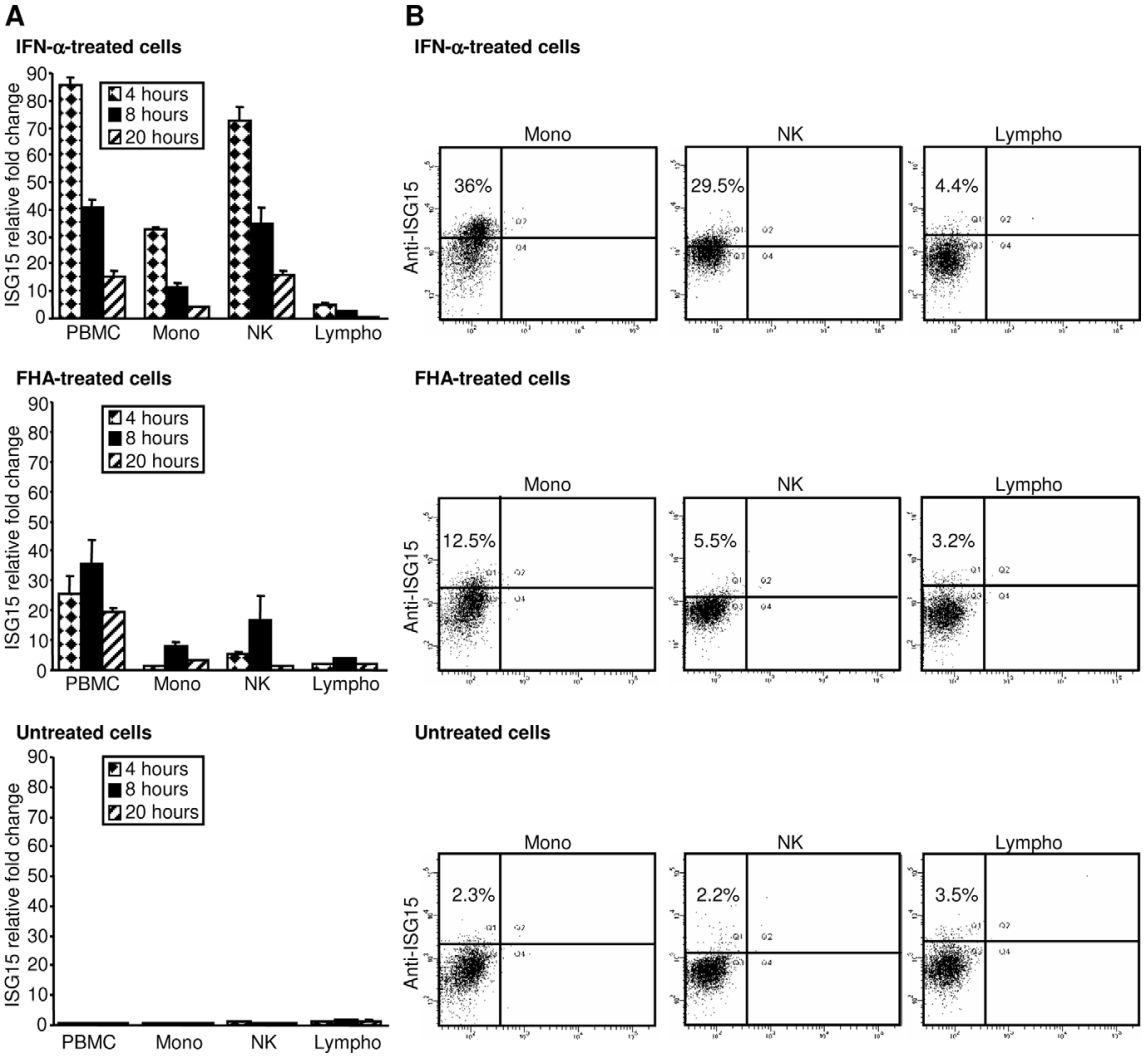
ISG15 mRNA expression and intracellular protein production in PBMCs and cell subsets. PBMCs were separated into monocytes (Mono), lymphocytes (Lympho), and an NK-enriched cell population (NK), using magnetic beads. (A) For ISG15 mRNA expression, 2×10^6^ cells/ml were cultured for 4, 8, and 20 hrs in the presence of IFN-α (IFN-α-treated cells) at 1,000 U/ml or FHA-2 (FHA-treated cells) at 5 µg/ml or without treatment. At each time point, total RNA was extracted and analyzed by RT-PCR. The fold expression change shown in the Figure is relative to levels in untreated cells at the beginning of the time course; the standard deviations for duplicate measurements are displayed. (B) For intracellular detection of ISG15, monocytes, NK-enriched cells, and lymphocytes were stimulated for 4 hrs with IFN-α (IFN-α-treated cells) at 1,000 U/ml or FHA-2 (FHA-treated cells) at 5 µg/ml or without treatment. Cells were then stained for intracellular ISG15 using the mouse monoclonal anti-ISG15 clone 4.1 labeled with AlexaFluor 647 (AF647). The number in the upper left corner of each graph represents the percentage of total cells testing positive for ISG15.

Next, to identify the PBMC subset type(s) that produced ISG15 in response to FHA, PBMCs were separated into monocytes, lymphocytes, and natural-killer (NK) cells. From each purified or enriched cell population, 2×10^6^ cells were stimulated with 1,000 U/ml IFN-α or 5 µg/ml FHA-2. ISG15 mRNA abundance was determined by real-time RT-PCR after 4, 8, and 20 hrs (Fig. 5A); intracellular levels of ISG15 protein were evaluated by FACS analysis after 4 hrs of stimulation (Fig. 5B).

As shown in Fig. 5A, IFN-α produced roughly comparable levels of ISG15 transcript in PBMCs and NK-enriched cells, with peak expression (85±3 and 73±5-fold respectively) at 4 hrs of treatment. Monocytes exhibited similar expression kinetics, but at a lower magnitude (32±1-fold after 4 hrs of treatment). Lymphocytes were the least responsive cells with a maximum of 5±1-fold change. The responsiveness to FHA treatment by the different cell types showed the same profile. As previously observed (Fig. 3), peak transcript levels of ISG15 were reached at 8 hrs post-treatment, instead of 4 hrs as observed in IFN-α-treated cells. Thus, among the mixed PBMC population, NK-enriched cells and monocytes, but not lymphocytes, induced expression of ISG15 in response to IFN-α and FHA stimulation. ISG15 expression was not induced in untreated cells during this experiment. Expression of intracellular ISG15 protein followed a similar pattern (Fig. 5B): after IFN-α stimulation, the fraction of ISG15-positive monocytes increased from 2.3% to 36% and NK cells from 2.2% to 29.5%, representing 15.6- and a 13.4-fold increases in ISG15-positive cells, respectively. The fraction of ISG15-positive lymphocytes was not affected by IFN-α treatment. Similarly, after 4 hrs of FHA treatment, 12.5% and 5.5% of the monocytes and NK cells, respectively, produced ISG15, while only 2.3% and 2.2% of the corresponding untreated cells expressed ISG15. The fraction of ISG15-positive lymphocytes remained unchanged. These results suggested that monocytes and NK cells are the major producers of ISG15 upon exposure of PBMCs to FHA.

## Discussion

Microbial adherence factors sometimes provoke complex signalling events and downstream biological processes in host cells following initial attachment to their target. This appears to be the case for the dominant *B. pertussis* adhesin, FHA, which is secreted in substantial quantities by this respiratory pathogen and which appears to express a variety of immunomodulatory activities [12]–[14], [17]. In this study, we analyzed the genome-wide gene expression responses of human PBMCs to purified FHA, in an effort to appreciate better the range of activities associated with this protein. PBMCs were selected because they encompass a diverse repertoire of both innate and adaptive immune functions and because of their roles in surveillance for infectious threats, both directly, through contact with infectious agents, and indirectly, through interactions with infected cells and tissues by means of secreted signalling molecules [47]. Moreover, a previous study investigated the effect of heat-killed *B. pertussis* on human PBMCs at the genome wide level [20]. Our data highlight the broad range of transcripts whose expression is affected by this bacterial adhesin. Of interest, our study revealed significant changes in the expression of several IFN-regulated genes, including those associated with the type I IFN response and with ISGylation.

We employed FHA preparations, purified using two different methods from four *B. pertussis* strains, in order to recognize conserved features of this protein and avoid strain-specific biases. The four strains were chosen to represent both recent clinical isolates and laboratory-adapted strains. One of the FHA preparations has been previously used to demonstrate FHA pro-inflammatory and pro-apoptotic activities, as well as its ability to modulate the NF-κB pathway [12], [13]. In addition, we reasonably postulated that, if contaminants should be co-purified with FHA, they would very likely be different and be present in different amounts in the various preparations, leading to a heterogenous response. Thus, our use of different preparations of FHA and the finding of similar, if not identical, gene expression patterns induced by them in host cells provides evidence of biological reproducibility in the data and suggests functional homogeneity among FHA preparations.

FHA altered the expression of 1,235 elements, representing 683 known unique genes. For most of these genes (419/683), their expression was up-regulated, suggesting that FHA more often activates than represses the host cell processes at the transcriptional level. All but one of the 12 genes that were part of a “common host-transcriptional response” to bacteria mediating inflammation [21] were also up-regulated in our data set; this finding highlights the important contribution of cell-free FHA to the inflammatory process associated with *B. pertussis* and with whooping cough [48] since a single virulence factor can induce a specific set of genes often reported as up-regulated by whole bacteria, viruses, yeast, protozoa, and helminths.

The cytokines induced by FHA might indirectly promote *B. pertussis* binding to host cells; IL-8, SCYA2/CCL2, GRO1/CXCL1, TNF-α, and IFN-γ, all induced at least at the transcriptional level by FHA (and in some cases, shown at the level of secreted protein), are known to promote expression of CD11b/CD18 (CR3) [49]–[51] which may serve as a receptor for FHA at the surface of neutrophils and monocytes [9], [52]. FHA might also influence host cell-cell interactions by up-regulating ICAM1 at the surface of PBMCs, similar to the process that has been observed with *Mycobacterium tuberculosis* [53] and *Streptococcus pneumoniae* pneumolysin [54]. Taken together, these results suggest that, in addition to acting as an adhesin, FHA influences host cell-cell interactions and inflammatory responses. *B. pertussis* FHA also affects the nature and the magnitude of the immune response that develops during *Bordetella* infection, similar to the effects reported for *B. bronchiseptica* FHA [17].

In addition to activating a common host transcriptional response, FHA also induced a more specific transcriptional program, not seen previously with heat-killed *B. pertussis* or *B. pertussis* LPS treatment (FHA-activation cluster). There are at least three reasons why this response may be specific to FHA and not due to LPS contamination. First, all four FHA preparations, independent of strain background, method of purification, and amount of contaminating LPS (<0.2–0.45 µg/ml), induced reproducible and consistent gene expression profiles. Second, by comparison with Boldrick's data set [20], we identified distinct gene expression profiles associated with BpeLPS that were not associated with FHA in our experiments. Third, LPS with endotoxin activity equal to that observed in our FHA preparations has been shown to induce a different TNF-α response [13]. We do not believe that the trace amount of adenylate cyclase (AC) toxin present in each FHA preparation (≤0.0135 pmol/ml, corresponding to approximately 2.4 ng/ml) plays any role in this observed host response. Indeed, only high concentrations (20 ng/ml) were recently used by Cheung *et al.* to evaluate the transcriptional responses of murine macrophages to *B. pertussis* AC toxin [55] and none of the genes present in our “FHA-activation cluster” showed any response in the Cheung study. In addition, the concentrations of LPS found in our FHA preparations are below the amount needed for intracellular calcium rise in J774A.1 and CHO cells [56], for proliferation/IL-2 secretion from peripheral blood lymphocytes [57], for inducing cytotoxicity in J774 cells, and for increased cAMP production in J774 cells [58]. The reason for obtaining a FHA-specific transcriptional program not observed after treatment with heat-killed *B. pertussis*, might simply be explained by the fact that, in our study, the amount of FHA used to treat the cells is higher than the amount of FHA present in a bacterial suspension. Alternatively, FHA might have been affected by the heat treatment, supported by the fact that heat-inactivation of FHA suppress its ability to agglutinate red blood cells. A third explanation could be that, in the presence of other bacterial components (such as those in a bacterial suspension), the transcriptional changes induced by FHA might be diminished by other bacterial components.

The most notable feature of this FHA-specific activation cluster was that 69% (9/13) of the identified genes are known to be regulated by IFN. Moreover, 18.3% (125 of 683 known unique genes) of all FHA-responsive genes are IFN-regulated. Among these were the genes encoding the major antiviral proteins PKR, OAS, MXl, MX2, ISG20, GBP1, PML, ADAR1, and ADA; this finding suggested that FHA activates a host IFN type I response. In fact, FHA induces IFN-α secretion by PBMCs. While *B. pertussis* is known to induce the production of IFN-γ [59], the expression of a type I IFN (IFN-β) has only recently been reported, in human monocyte-derived dendritic cells infected with an adenylate cyclase-deficient *B. pertussis* strain [60]. To our knowledge, our work is the first to reveal induction of a strong IFN and IFN type I response by FHA, and one of the first to report a type I IFN response to a secreted bacterial virulence factor.

Type I IFNs are potent antiviral immunoregulators that also play a fundamental role in the host response to bacterial infections [36]. Several bacteria induce production of type I IFNs by infected cells or in infected mice; in the case of *L. monocytogenes*, activation of type I IFN signaling induces apoptosis of splenic cells, which contributes to the virulence of the infection. Because FHA-induced apoptosis in human cells is only partly dependent on the production of TNF-α [13], we hypothesize that the FHA-induced type I IFN response might contribute to cell death. It might thus represent an additional mechanism for rendering the host more susceptible to infection.

An intriguing feature of the FHA-induced IFN type I response is the upregulation of several members of the ISGylation pathway: the ubiquitin-like protein ISG15, its specific isopeptidase USP18, and the ISG15-activating enzyme UBE1L. ISG15 is rapidly and strongly induced in response to stimulation by type I IFN [61] and by certain types of LPS [62]. In human PBMCs, ISG15 transcript abundance increases after stimulation with heat-killed *E. coli*, but not *S. aureus*, *B. pertussis*, or *B. pertussis* LPS [20]. Although we confirmed that BpeLPS was only a weak inducer of ISG15 mRNA, we demonstrated that purified FHA induced high levels of ISG15 mRNA, as well as increased levels of free ISG15 and several ISGylated conjugates. The presence of an additional conjugate of ∼130 kDa in FHA-treated cells suggests that FHA might modify the function of at least one target protein in the host. However, because multiple molecules of ISG15 may conjugate to the same protein [26], the actual size of the target protein may be difficult to predict *a priori*; further investigation will be needed to identify the targets of ISGylation resulting from FHA exposure.

Our study demonstrated that monocytes and an NK-enriched cell population were, in contrast to lymphocytes, the main producers of ISG15, both at the mRNA and protein levels. These results not only are consistent with earlier findings [63], but they also reveal that NK cells are not only the target of ISG15 [63], but also one of the major producers of ISG15, suggesting autocrine regulation of ISG15 in NK cells. This seems to be a feature of the ISGylation pathway, independent of the stimulus.

The peak levels of ISG15 (and USP18) transcript abundance were consistently observed after 8 hours of FHA exposure, rather than after 4 hours, as is the case after EcLPS and IFN-α treatment. These findings suggested two hypotheses. First, FHA might delay, and then amplify the expression of ISG15 and USP18 through typical pathways and mechanisms. Alternatively, FHA might use a different signaling pathway that results in delayed expression of these genes. In any case, the late induction of ISG15 expression could be responsible for the late inhibition of NF-κB activation [12] due to the negative regulation of this pathway by ISGylation [64].

ISG15 interferes with ubiquitination of viral proteins [31], [32] and exhibits cytokine-like immunomodulatory activities [45], [46]. In addition, USP18 was recently identified as a novel regulator of IFN signaling [42]. Even though it is now clear that ISG15 has the capacity to modulate diverse cellular and physiologic functions, the roles of ISG15 and the ISGylation pathway in host defense against infectious agents have not been fully elucidated [65]. We believe that our work, by broadening our awareness of the involvement of this pathway in bacterial infection, might contribute to an understanding of the “ubiquitin-like enigma” [24], and help suggest mechanisms by which bacterial pathogens modulate the host immune response.

## Materials and Methods

### Bacterial strains, bacterial products, and culture conditions

Clinical isolates, laboratory strains, and isogenic mutant strains of *B. pertussis* (Table 1) were cultured in modified Stainer-Sholte medium (SSM) [66] or on Bordet-Gengou (BG) agar (Difco Laboratories, Detroit, MI) plates supplemented with 13% vol/vol whole sheep blood (Microbiological Media, Concord, CA) in the presence of antibiotic(s) when appropriate. *E. coli* Sm10λ-pir [67] was grown in Luria Bertani (LB) broth or on LB agar plates. Antibiotics (Sigma, St Louis, MO) were used at the following concentrations: ampicillin, 200 µg/ml; nalidixic acid, 50 µg/ml; rifampicin, 30 µg/ml; streptomycin, 100 µg/ml; gentamicin, 50 µg/ml. *E. coli* O111:B4 LPS was purchased from Sigma, and *B. pertussis* LPS was a generous gift from A. Preston (Department of Molecular and Cellular Biology, University of Guelph, Ontario, Canada).

**Table 1:** *B. pertussis* Strains Used in This Study.

Name	Parental strain	Genotype or Relevant Characteristic	Reference/Source
Bpe52		Clinical isolate (1994), Gm^R^, Rif^R^	[83]
BP338	Tohama I	Lab strain (1983), Gm^R^, Nal^R^	[84]
Minnesota 1		Clinical isolate, Gm^R^, Rif^R^	[20]
Bpe136	BP338	Gm^R^, Nal^R^, Sm^R^	This work
Bpe144	Minnesota 1	Gm^R^, Rif^R^, Sm^R^	This work
Bpe146	Bpe52	Gm^R^, Rif^R^, Sm^R^	This work
Bpe160	Bpe136	Gm^R^, Nal^R^, Sm^R^, Δ*ptx*-*ptl*	This work
Bpe162	Bpe144	Gm^R^, Nal^R^, Sm^R^, Δ*ptx*-*ptl*	This work
Bpe163	Bpe146	Gm^R^, Nal^R^, Sm^R^, Δ*ptx*-*ptl*	This work

Rif: rifampicin; Nal: nalidixic acid; Sm: streptomycin; Gm: gentamicin.

### Construction of *B. pertussis* Δ*ptx-ptl*

To prevent co-purification of pertussis toxin with FHA, the entire *ptx-ptl* region was deleted from Bpe136 (Sm^R^ derivative of BP338), Bpe144 (Sm^R^ derivative of Minnesota 1), and Bpe146 (Sm^R^ derivative of Bpe52) by homologous recombination, as previously described [68]. Plasmid pDMC28 (a generous gift from D. L. Burns, Division of Bacterial, Parasitic, and Allergenic Products, Center for Biologics Evaluation and Research, Food and Drug Administration, Bethesda, Maryland, USA) was transformed into *E. coli* Sm10**λ**-pir. Allelic exchange was performed on BG-agar plate for 4 hours between *E. coli* Sm10**λ**-pir (pDMC28) and *B. pertussis* (Bpe136, Bpe144, or Bpe146). Co-integrants were selected for their resistance to both gentamicin and nalidixic acid or rifampicin, and their sensitivity to streptomycin. Loss of the plasmid by homologous recombination was selected by sequentially plating the colonies on BG agar containing streptomycin, and then BG agar containing gentamicin. The absence of the *ptx* and *ptl* genes was confirmed by PCR using primers annealing at positions 3991407–3991430 and 3991407–3991430 of the assembled *B. pertussis* sequences from the Sanger Centre (www.sanger.ac.uk), between *ptlA* and *ptlB*. Absence of pertussis toxin was verified by Western-blot using a monoclonal antibody against the S1 subunit (m1B7, a generous gift from J. Maynard, Department of Chemical Engineering and Materials Science, University of Minnesota, MN, USA) (data not shown).

### Cell separation and culture

Human PBMCs were purified from freshly drawn blood or from the “buffy coat” fraction from healthy donors (Stanford Blood Center, Stanford, CA), using Ficoll-Paque (Amersham Biosciences, Piscataway, N.J.) according to the manufacturer's instruction. PBMCs were resuspended in RPMI-1640 medium (American Type Culture Collection, ATCC, Manassas, VA) supplemented with 10% fetal bovine serum (ATCC), 100 U/ml penicillin, and 100 µg/ml streptomycin (Gibco, Invitrogen Corportation, Carlsbad, CA).

For some experiments, PBMCs were further separated into natural killer (NK) cells, monocytes, and lymphocytes using magnetic cell separation (Miltenyi Biotec, Auburn, CA). NK cells were first positively enriched from PBMCs using the CD56 MicroBeads kit. The monocytes and lymphocytes present in the flow-through were further separated using the Monocyte Isolation Kit II; the resulting CD14+ cells were 81% pure. (CD14 antigen is expressed at high levels on monocytes. In addition, anti-CD14 antibody recognizes interfollicular macrophages, reticular dendritic cells, and some Langerhans cells [BD Biosciences]). CD3+ and CD20+ cells were 86% pure. (The anti-CD3 antibody recognizes a major subset of peripheral blood lymphocytes, but not monocytes or granulocytes [BD Biosciences]. CD20 phosphoprotein is found on circulating peripheral blood B lymphocytes [BD Biosciences].) The NK-enriched fraction contained 52% CD16+ cells, as evaluated by FACS analysis. (CD16 is expressed on NK cells, as well as on macrophages and granulocytes [BD Biosciences].)

### FHA purification

FHA was purified, as previously described [69], from *B. pertussis* Bpe160, Bpe162, and Bpe163 and named FHA-2, FHA-3, and FHA-4, respectively. Briefly, *B. pertussis* liquid cultures were harvested when the optical density at 600 nm was between 2.5 and 4. The culture supernatant was supplemented with Complete Protease Inhibitor (Roche, Indianapolis, IN) and applied at 2 ml/min on a 5 ml HiTrap Heparin column (Amersham Biosiciences). Purification was performed using an FPLC system equipped with a P-500 pump and a FRAC-P100 fraction collector (Pharmacia LKB). FHA was eluted at 2 ml/min with 1× PBS, 0.5 M NaCl (“Mock-2”). Total protein concentrations, determined by the BCA test (Pierce, Rockford, IL), were 560, 300, and 340 µg/ml for FHA-2, FHA-3, and FHA-4, respectively. FHA-1 (a kind gift from M. Pizza and R. Rappuoli, Novartis Vaccines, Siena, Italy) was isolated from *B. pertussis* Wellcome 28 (W28) [70] using Matrex Cellufine Sulfate (Millipore) and stored in a solution containing 50% glycerol, 0.5 M NaCl, and 25 mM Na_2_HPO_4_ (“Mock-1”). The in-house FHA preparations (FHA-2, -3, and -4) were evaluated for the presence of adenylate cyclase (AC) toxin using ELISA [71], and for the presence of LPS using the purpald assay [72] (data not shown). Trace amounts of AC (≤2.7 pmol/mg of total protein) and LPS (≤27 µg/ml) were found in all of the FHA preparations. After dilution of the FHA preparations into cell cultures to obtain the working concentration (5 µg/ml), the maximum effective LPS and AC concentrations during our experiments never exceeded 0.45 µg/ml and ≤0.0135 pmol/ml respectively. Purity of the FHA preparations was evaluated by Coomassie blue staining and Western-blot analysis using anti-FHA antibodies. Functionality of the FHA preparations was investigated using a hemagglutination assay and was found to be retained as compared to both heat-inactivated and proteinase-K-treated FHA preparations (Fig. S1). The FHA-1 preparation was used as a reference, as it was previously shown to induce both pro-inflammatory and pro-apoptotic responses in human cells [13] and to modulate the NF-κB pathway [12].

### cDNA microarray and hybridization

Total RNA was extracted from PBMC using Trizol-LS (Invitrogen), and then amplified using the MessageAmp aRNA Kit (Ambion, Austin, TX) according to the manufacturer's instructions. Fluorescently labelled cDNA was hybridized to a human cDNA microarray [73] in a two-color comparative format, with the experimental samples labelled with Cy5 (Amersham Biosciences) and a reference pool of mRNA (Universal Human Reference RNA, Stratagene, La Jolla, CA) labelled with Cy3 (Amersham Biosciences). The array used for these studies contained 37,632 spots derived from cDNA clones representing approximately 18,000 unique human genes and was previously used in a variety of published studies [39], [74]–[78].The labelling reaction, and microarray hybridization and washing were performed as previously described (http://cmgm.stanford.edu/pbrown/protocols/4_human_RNA.html), with the following modifications: we used 4 µg of amplified RNA to anneal with 10 µg pd(N)6 random hexamers (Amersham Biosciences), and hybridization was performed for 16 hours at 65°C.

### Data filtering and analysis

Arrays were scanned using a GenePix 4000b microarray scanner (Axon Instruments, Foster City, CA), and image analysis was performed using GenePix Pro version 5.0 (Axon Instruments). Data were expressed as the log_2_ ratio of background-subtracted fluorescence intensities from the sample versus the reference, for each element on the array [79]. Data were filtered to retain only the elements with a signal/background intensity ratio >2.5 (in either sample or reference channel) in at least 80% of the 23 arrays, and with a regression correlation coefficient between sample and reference signal >0.6. Using these criteria, we identified 25,867 well-measured elements. The time course data from each treatment were normalized to the averaged time-zero data of four replicates. The data were hierarchically clustered using the CLUSTER program [80], and displayed using TREEVIEW (http://rana.lbl.gov/EisenSoftware.htm).

### Real-time reverse transcription (RT)-polymerase chain reaction (RT-PCR)

Total RNA was purified using the RNeasy mini kit (QIAGEN, Valencia, CA), and 200 ng was submitted to cDNA synthesis using TaqMan RT reagents (Applied Biosystems [ABI], Foster City, CA) followed by PCR using the Taqman expression assays (ABI) for USP18, ISG15, and 18S rRNA according to the manufacturer's instructions on the ABI Prism 7900HT sequence detection system (ABI). At the beginning of each time course experiment (time zero), total RNA from untreated cells (two or three independent wells) was also extracted and used as the reference for comparison with the expression level of treated cells using the comparative C_T_ method [81]. Within each experiment, the PCR product of each sample was measured in duplicate; the mean, as well as the range of fold change are displayed on the graph.

### Western-blot analysis

A total of 5×10^7^ PBMCs were stimulated with 1,000 U/ml IFN-α (Biosource, Camarillo, CA), 1 µg/ml EcLPS, 10 µg/ml BpeLPS, or 5 µg/ml FHA-2, or left untreated for 20 hours. Cell lysates were prepared according to a protocol adapted from D'Cunha et al. [82]. PBMCs were washed and resuspended into 500 to 800 µl cold lysis buffer (0.02 M Tris-Cl pH 7.5, 0.15 M NaCl, 0.5% deoxycholate, 0.5% triton X-100, 0.05% SDS, 0.01 M EDTA, and 1× solution of Complete Protease Inhibitor [Roche]). After incubation on ice for 30 min, cells were passed through a 21-gauge needle and centrifuged for 20 min at 12,000×*g* at 4°C. The protein concentration was determined using MicroBCA (Pierce), and 90 µg were loaded onto 10% (for separation of ISGylated proteins) or 15% (for separation of free ISG15) SDS-polyacrylamide gels and transferred to Immobilon-P PVDF membrane (Millipore Corporation, Bedford, MA). ISGylated proteins were detected with a 1/500 dilution of rabbit-anti-human ISG15 IgG (Rockland, Gilbertsville, PA); free ISG15 was detected using a 1/1,000 dilution of mouse monoclonal anti-human ISG15 (clone 2.1; a kind gift from E. Borden, Cleveland Clinic Foundation, Cleveland, OH). Anti-human ISG15 antibodies were detected with HRP-conjugated goat-anti rabbit (1/10,000, Sigma), or with anti-mouse (1/5,000, Sigma) antibodies. Blots were visualized using the ECL-Plus Western blotting detection kit according to the manufacturer's instructions (Amersham Biosciences). To assess equal protein loading, membranes used for the detection of free ISG15 were stripped and re-probed with a 1/1,000 dilution of mouse monoclonal anti-actin antibody (Lab Vision Corporation, Fremont, CA) and a 1/5,000 dilution of anti-mouse secondary antibody (Sigma). As a positive control, 20 ng human recombinant ISG15 (BostonBiochem, Cambridge, MA) was loaded on all gels. The higher molecular weight of recombinant ISG15 compared to endogenous ISG15 is due to an extra amino acid left after cleavage of a GST tag from the recombinant protein.

### Cytokine measurement by ELISA and Luminex assay

Detection of IFN-α in the supernatant of cultured PBMCs was performed using the Human Interferon Alpha (Hu-IFN-α) ELISA kit (Biosource International, Inc., Camarillo, CA) according to the manufacturer's instructions. Each sample was measured twice in duplicate and the standard deviation was calculated based on the average value of the two independent measurements.

Samples with supernatant were sent to an outside laboratory, Upstate (Charlottesville, VA) for measurements of TNF-α, IL-1β, IL-6, and IL-8 concentration using the Beadlyte Human Cytokine Profiler Analysis (Upstate). The results reported were the average of triplicate measurements.

### FACS analysis

Flow cytometry was performed at the Stanford University Digestive Disease Centre Core Facility (VA Hospital, Palo Alto, CA, USA) using the BD LSR II system (BD Biosciences, San Jose, CA). For each sample, a minimum of 100,000 events were collected. Data were analysed with FACSDiva software (BD Biosciences).

For cell surface staining, approximately 1×10^6^ cells were washed and incubated with 10% human serum before staining with one of the following FITC- or PE-conjugated antibodies (Caltag Laboratories, Burlingame, CA, and BD PharMingen): CD3, CD20, CD14, and CD16. After staining, cells were washed once and fixed with 1% paraformaldehyde.

For the detection of intracellular ISG15, cells were incubated for 4 hours with stimulus, and in the presence of 10 µg/ml brefeldin A (Sigma) during the last 2 hours. Approximately 1×10^6^ cells were washed, resuspended in 10% human serum for 20 min on ice, washed, and resuspended in 2% paraformaldehyde. After 20 min incubation on ice, cells were washed and resuspended in PBA buffer (0.2% BSA and 0.09% sodium azide in 1× PBS). After overnight incubation at 4°C, cells were resuspended in 10 µl saponin buffer (0.2% BSA and 0.5% saponin in 1× PBS) containing 10 µg mouse IgG (Jackson ImmunoResearch, West Grove, PA) to block nonspecific binding; Alexa Fluor 647-conjugated mouse monoclonal anti-ISG15 or Alexa Fluor 647-conjugated mouse IgG antibodies were then added to monitor nonspecific binding. After 15 min incubation on ice and washing with cold saponin buffer, cells were resuspended in 500 µl PBA buffer for analysis. Alexa Fluor 647-conjugated antibodies were obtained by labelling 100 µg of mouse monoclonal anti-ISG15 antibody (clone 4.1, a generous gift from Dr. E. Borden, Cleveland Clinic Foundation, Cleveland, OH) and 100 µg of purified mouse IgG1 (eBioscience, San Diego, CA) using the Alexa Fluor 647 monoclonal antibody labelling kit (Molecular Probes, Eugene, OR) according to the manufacturer's instructions.

### Microarray data deposition

The data discussed in this publication have been deposited at NCBI Gene Expression Omnibus (GEO, http://www.ncbi.nlm.nih.gov/geo) and are available through GEO Series accession number GSE8802.

## Supporting Information

Figure S1 **Integrity and functionality of FHA purified from** ***B. pertussis*** **culture supernatants.** FHA was purified from *B. pertussis* culture supernatant as described in Materials and Methods. **A.** Total protein concentration in FPLC elution fractions 4–8 of Bpe160 (FHA-2), Bpe162 (FHA-3), and Bpe163 (FHA-4). **B.** Coomassie blue staining of Bpe160 (160) and Bpe163 (163) liquid culture supernatants (loaded with the equivalent of 400 µl liquid culture at OD600 nm = 3), as well as their corresponding purified, FHA-2 (10 µl fraction #5) and FHA-4 (34 µl of fraction #5). Protein size in kDa of the molecular weight markers (MW) is indicated on the left. **C.** Western-blot analysis of FHA-1 using anti-FHA antibody (M08), with protein size (kDa) indicated on the right. **D.** Agglutination with FHA-1, as well as FHA-2 (elution fractions #9 (193 µg protein/ml), #10 (1200 µg protein/ml), #11 (821 µg protein/ml), and #12 (373 µg protein/ml) from a purification similar to that shown in A) was performed by adding 50 µl FHA to 50 µl 1% sheep blood and incubating for 1 h at 37°C in a V-shape bottom 96-well plate. PBS was used as negative control and did not agglutinate the red blood cells. FHA-1 was either heat-inactivated for 20 minutes at 95°C (heat) or incubated with proteinase-K (Prot. K), or left untreated (WT) before incubation with red blood cells.
(TIF)

Table S1 **FHA-activated elements.** The 817 elements whose expression was activated by at least 3-fold in FHA-treated compared to untreated cells are represented, together with their expression values.
(XLS)

Table S2 **FHA-repressed elements.** The 418 elements whose expression was repressed by at least 3-fold in FHA-treated compared to untreated cells are represented, together with their expression values.
(XLS)

Table S3 **IFN-regulated genes whose expression is affected by FHA treatment.** The IFN-regulated genes were selected from the 1,235 FHA-responsive elements by comparison with lists of known IFN-α-, -β-, and -γ-regulated genes [36]–[40]. This comparison returned 296 elements.
(XLS)
