# Supplementary figures and images for: Modulation of the Host Interferon Response and ISGylation Pathway by B. pertussis Filamentous Hemagglutinin
Source: PLoS One. 2011 Nov 30;6(11):e27535. doi: 10.1371/journal.pone.0027535 (PMC3227562; doi:10.1371/journal.pone.0027535)

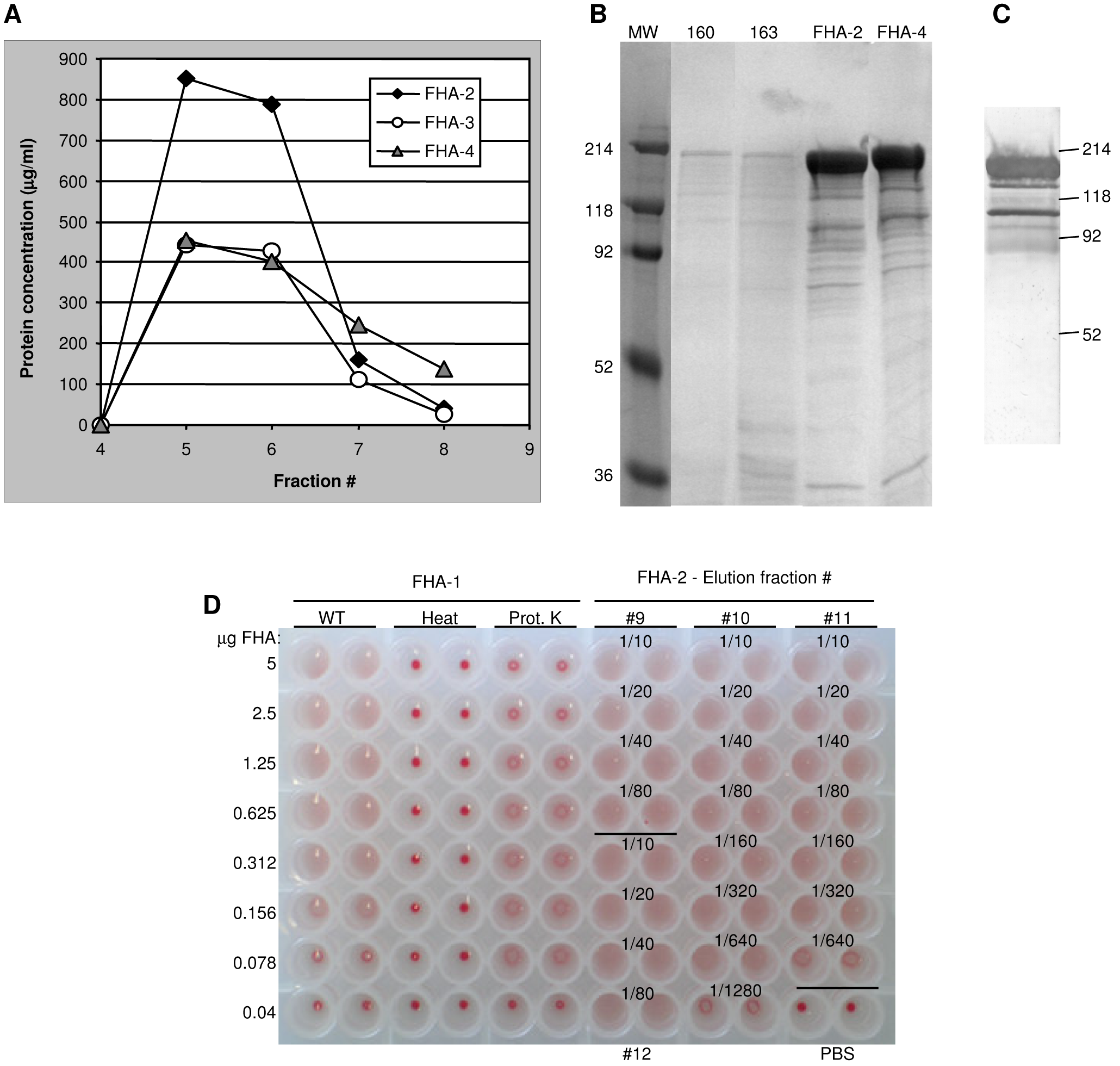

Supplement: Figure S1 — Integrity and functionality of FHA purified from B. pertussis culture supernatants. FHA was purified from B. pertussis culture supernatant as described in Materials and Methods. A. Total protein concentration in FPLC elution fractions 4–8 of Bpe160 (FHA-2), Bpe162 (FHA-3), and Bpe163 (FHA-4). B. Coomassie blue staining of Bpe160 (160) and Bpe163 (163) liquid culture supernatants (loaded with the equivalent of 400 µl liquid culture at OD600 nm = 3), as well as their corresponding purified, FHA-2 (10 µl fraction #5) and FHA-4 (34 µl of fraction #5). Protein size in kDa of the molecular weight markers (MW) is indicated on the left. C. Western-blot analysis of FHA-1 using anti-FHA antibody (M08), with protein size (kDa) indicated on the right. D. Agglutination with FHA-1, as well as FHA-2 (elution fractions #9 (193 µg protein/ml), #10 (1200 µg protein/ml), #11 (821 µg protein/ml), and #12 (373 µg protein/ml) from a purification similar to that shown in A) was performed by adding 50 µl FHA to 50 µl 1% sheep blood and incubating for 1 h at 37°C in a V-shape bottom 96-well plate. PBS was used as negative control and did not agglutinate the red blood cells. FHA-1 was either heat-inactivated for 20 minutes at 95°C (heat) or incubated with proteinase-K (Prot. K), or left untreated (WT) before incubation with red blood cells. (TIF) [file pone.0027535.s001.tif]
